# Development of structural colour in leaf beetles

**DOI:** 10.1038/s41598-017-01496-8

**Published:** 2017-05-02

**Authors:** Olimpia D. Onelli, Thomas van de Kamp, Jeremy N. Skepper, Janet Powell, Tomy dos Santos Rolo, Tilo Baumbach, Silvia Vignolini

**Affiliations:** 1 Department of Chemistry, University of Cambridge, Lensfield Road, Cambridge, CB2 1EW UK; 2 Laboratory for Applications of Synchrotron Radiation (LAS), Karlsruhe Institute of Technology (KIT), Kaiserstr. 12, D-76131 Karlsruhe, Germany; 3 CAIC, Anatomy Building, Cambridge University, Downing Street, Cambridge, CB2 3DY UK; 4 Institute for Photon Science and Synchrotron Radiation (IPS), Karlsruhe Institute of Technology (KIT), Hermann-von-Helmholtz-Platz 1, D-76344 Eggenstein-Leopoldshafen, Germany

## Abstract

Structural colours in living organisms have been observed and analysed in a large number of species, however the study of how the micro- and nano-scopic natural structures responsible of such colourations develop has been largely ignored. Understanding the interplay between chemical composition, structural morphology on multiple length scales, and mechanical constraints requires a range of investigation tools able to capture the different aspects of natural hierarchical architectures. Here, we report a developmental study of the most widespread strategy for structural colouration in nature: the cuticular multilayer. In particular, we focus on the exoskeletal growth of the dock leaf beetle *Gastrophysa viridula*, capturing all aspects of its formation: the macroscopic growth is tracked via synchrotron microtomography, while the submicron features are revealed by electron microscopy and light spectroscopy combined with numerical modelling. In particular, we observe that the two main factors driving the formation of the colour-producing multilayers are the polymerization of melanin during the ecdysis and the change in the layer spacing during the sclerotisation of the cuticle. Our understanding of the exoskeleton formation provides a unique insight into the different processes involved during metamorphosis.

## Introduction

In contrast to colours due to pigments, structural colouration originates from the interaction of light with sub-micrometer structured materials^1^. Examples of such striking and brilliant colourations are found in many different groups of organisms, including bacteria^2^, protists^3^, plants^4^, and animals^5^. The variety of colour-producing photonic mechanisms is tremendous - ranging from one- to three-dimensional systems. These structures can be highly periodic (where the long-range correlations between the elements give rise to bright metallic colourations^6^), partially disordered (here, the short-range correlations allow matte, isotropic colours^7^), or completely random (the absence of correlation between the scattering centres provides brilliant white reflectivity^8, 9^). Having evolved over more than 500 million years^10–12^, structural colours have been proven to play a key role in animal communication^13^, mating^14^, and camouflage^15^.

Such photonic structures are frequently found among arthropods and are particularly common in insects^16^. In fact, a great number of different species show structurally coloured scales^15^ or setae^17^. The most common and best understood colour-producing mechanism by far is the multilayer reflector, which is often found in beetles (Coleoptera). These reflectors may be located at different depths within the cuticle^18, 19^, which forms a multilayered exoskeleton^20^.

Being a natural fibre composite, the insect cuticle consists of chitin microfibrils embedded in a proteinous matrix^21, 22^ which usually contains pigments such as tannins or melanin. In many iridescent insects, multilayer reflectors are generated by stratified deposition of pigments in different cuticular layers. Depending on the pigments types and contents, the refractive index of pigmented layers can vary significantly with respect to the chitin-protein matrix^23^.

It has long been known that the cuticle is secreted by a single-sheet epithelium^24^ whose products can vary in time during the development, giving overlying layers: the outermost epicuticle and the underlying, thicker procuticle, which can be further divided into exocuticle and endocuticle^21^. The development of the trabeculae (i.e. the columns connecting top and bottom sides of the elytra) has also been investigated and reported to begin from the dorsal cuticle and progress towards the ventral cuticle and eventually merge with it^25^. However, little is known about the development of the photonic structures: previous studies have concentrated on the development of butterfly scales^26–29^ and bird feathers barbs^30^ showing the interplay of self-assembly and biologically-driven development. Even though the literature on metallic colouration in beetles is extensive, describing standard multilayer reflectors^31^, circularly polarising helicoids^32^, chirped broadband reflectors^18^, and their taxonomic distribution^33, 34^, their formation and development has never been investigated in living specimens. Employing a hierarchical approach, we describe the formation of a multilayer reflector in the exocuticle of developing European green dock leaf beetles (*Gastrophysa viridula*): from the observation of the macroscopic features to the investigation of micro-sized architectures via synchrotron microtomography (SR-*μ*CT) down to the nanoscale imaged by transmission electron microscopy (TEM). To complement the results, optical characterization of the developing exoskeleton is carried out on the cuticle of living specimens. The collected spectra for different stages of growth are then compared to computational simulations based on the transfer-matrix method^35^.

## Results and Discussion

The life cycle of *G*. *viridula* generally consists of the following phases: egg, three larval stages, pupa, and imago. In this study, we focus on the formation of the cuticular multilayer reflector in the hardened forewings (elytra) of the maturing adult beetle and refer to the following stages in the text: egg (1), third instar larva (2), young pupa (3), imago immediately after ecdysis (4), imago before cuticular expansion is completed (5), imago with fully developed cuticle (6). For a summary of the stages and relative imaging techniques, refer to Table 1.

**Table 1:** Summary of beetle’s life stages considered in this study.

Description	Age (/days)	Photo	SR-*μ*CT	TEM	Micrograph	Spectrum
1. Egg	0–7	1A				
2. Third instar larva	15–18	1B	2A, F			
3. Young pupa	18–20	1C	2B, G		4A	5A
4. Imago immediately after ecdysis	25–26	1D	2C, H	3A	4B	5A, B
5. Imago before cuticular expansion	26–27	1E	2D, I	3B	4C	5A, C
6. Imago with fully developed cuticle	30+	1F	2E, J	3C–F	4D	5A, D

**Figure 1:**
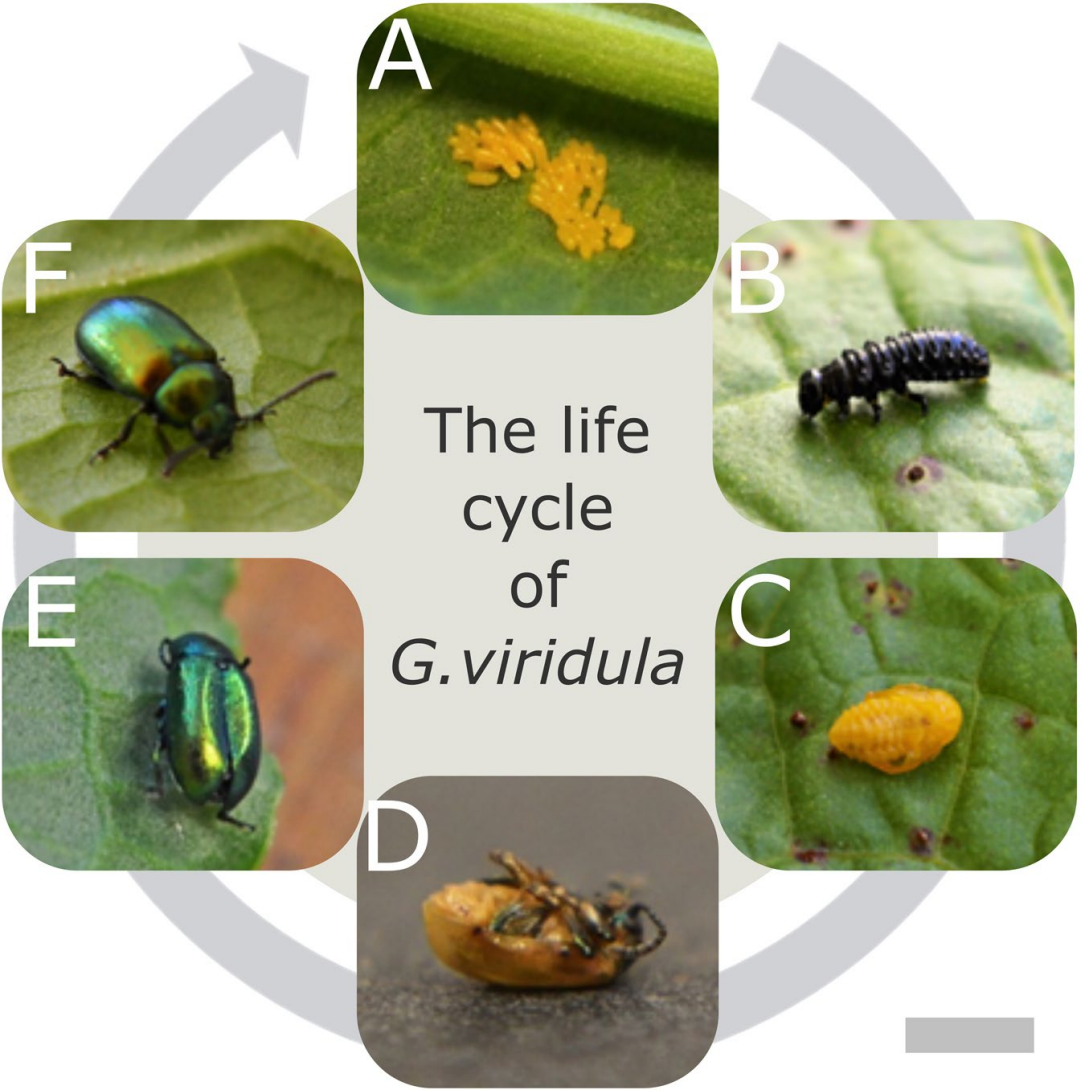
The life cycle of the *G*. *viridula* from the egg ((**A**) day 0–7) to the adult (**F**) via the larval stages ((**B**) days 8–18:), pupa ((**C**) days 18–25), after ecdysis ((**D**) day 25), and the final cuticle expansion (from (**E**) day 26). Scale bar: 1 cm.

The eggs (Fig. 1A) hatch 5–7 days after deposition. After feeding on dock leaves (*Rumex obtusifolius*) for 9–10 days, the larvae (Fig. 1B) molt and turn into yellow pupae (Fig. 1C, Supplementary Video 1) from which the imagoes emerge after 5–7 days of metamorphosis (Supplementary Video 2). The lifespan of the adult beetles varies between 20 and 30 days. Immediately after ecdysis, the cuticle of the imagoes is still largely yellow (Fig. 1D) and even one week later, the insects’ cuticles are not fully expanded (Fig. 1E) resulting in a weaker colouration compared to those of older adults (Fig. 1F).

**Figure 2:**
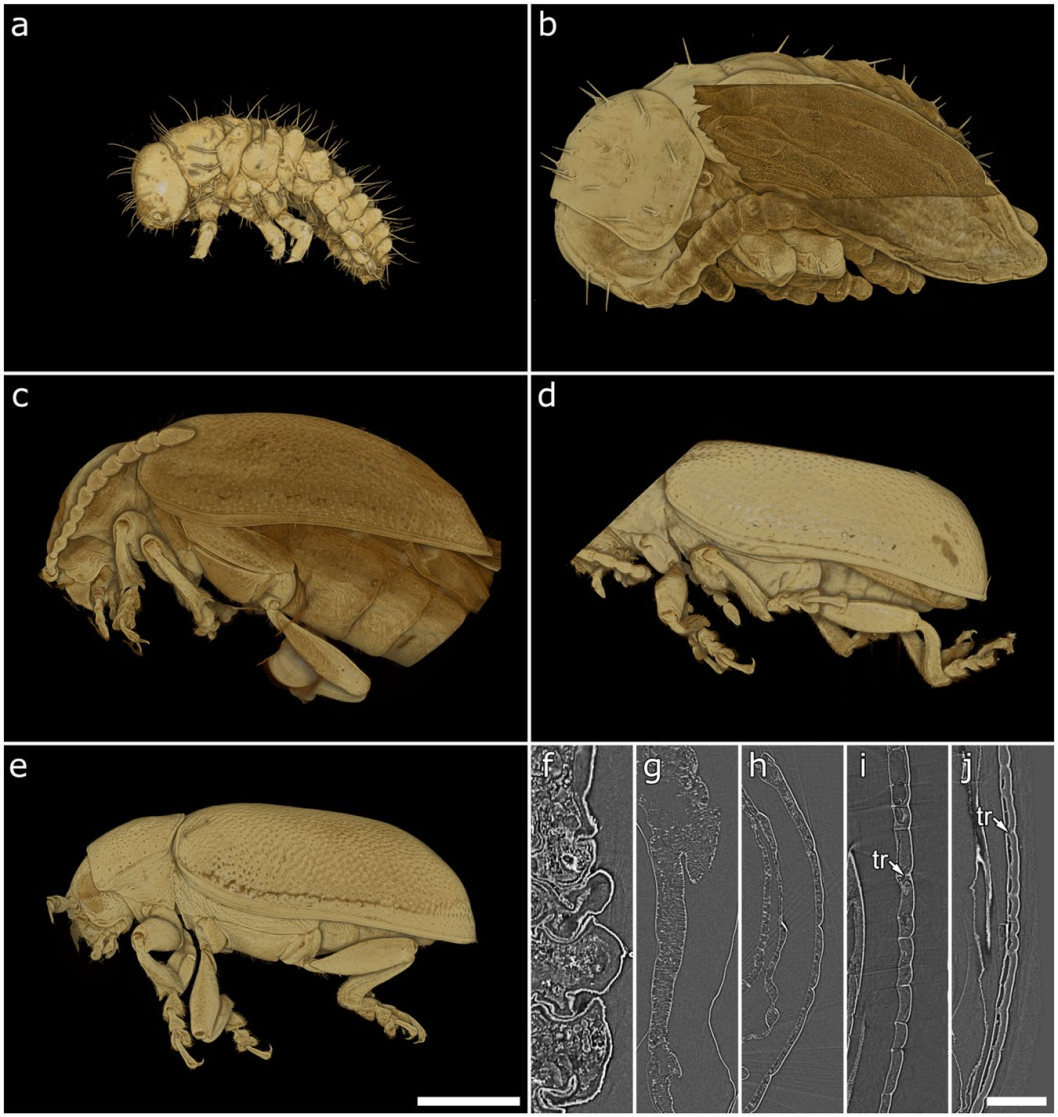
Results from SR-*μ*CT scans showing volume renderings (**a**–**e**) and cross-sections (**f**–**j**) of the respective dataset. 3rd instar larva, which exhibits a soft cuticle (**a**,**f**); young pupa (**b**,**g**), pupal skin partly digitally removed to reveal the developing elytron; imago immediately after ecdysis (**c**,**h**) the young imago (**d**,**i**), which shows a fully-formed yet rucked up cuticle and the formation of trabeculae (“tr” in the figure); old imago (**e**,**j**) with a fully developed cuticle. Scale bars: 1 mm.

In order to anatomically characterize the development of the beetles, we perform SR-*μ*CT scans of selected developmental stages (Fig. 2). The larvae do not exhibit wings but rather a soft, non-sclerotised cuticle (Fig. 2a,f). The elytra begin to form in stage 3 (Fig. 2b) and continue during stages 4 and 5 (Fig. 2c,d). However, the cuticle in stage 4 (Fig. 2h) is still considerably softer and thinner (1.0–1.5 *μ*m) than in the adults (4.0–4.5 *μ*m) in stages 5 and 6 (Fig. 2g,j). In stage 4, the trabeculae are seen to originate dorsally (Fig. 2h). It is only at a later stage that the trabeculae start merging with the ventral cuticle (Fig. 2i). Confirming the results in ref. 25, they are seen to have completely developed and thickened in the adult (Fig. 2j), spacing the inner and outer sections of the cuticle, which is about 4 *μ*m thick. Finally, in the fully developed adults (Fig. 2e) the cuticle is completely expanded (Fig. 2j).

**Figure 3:**
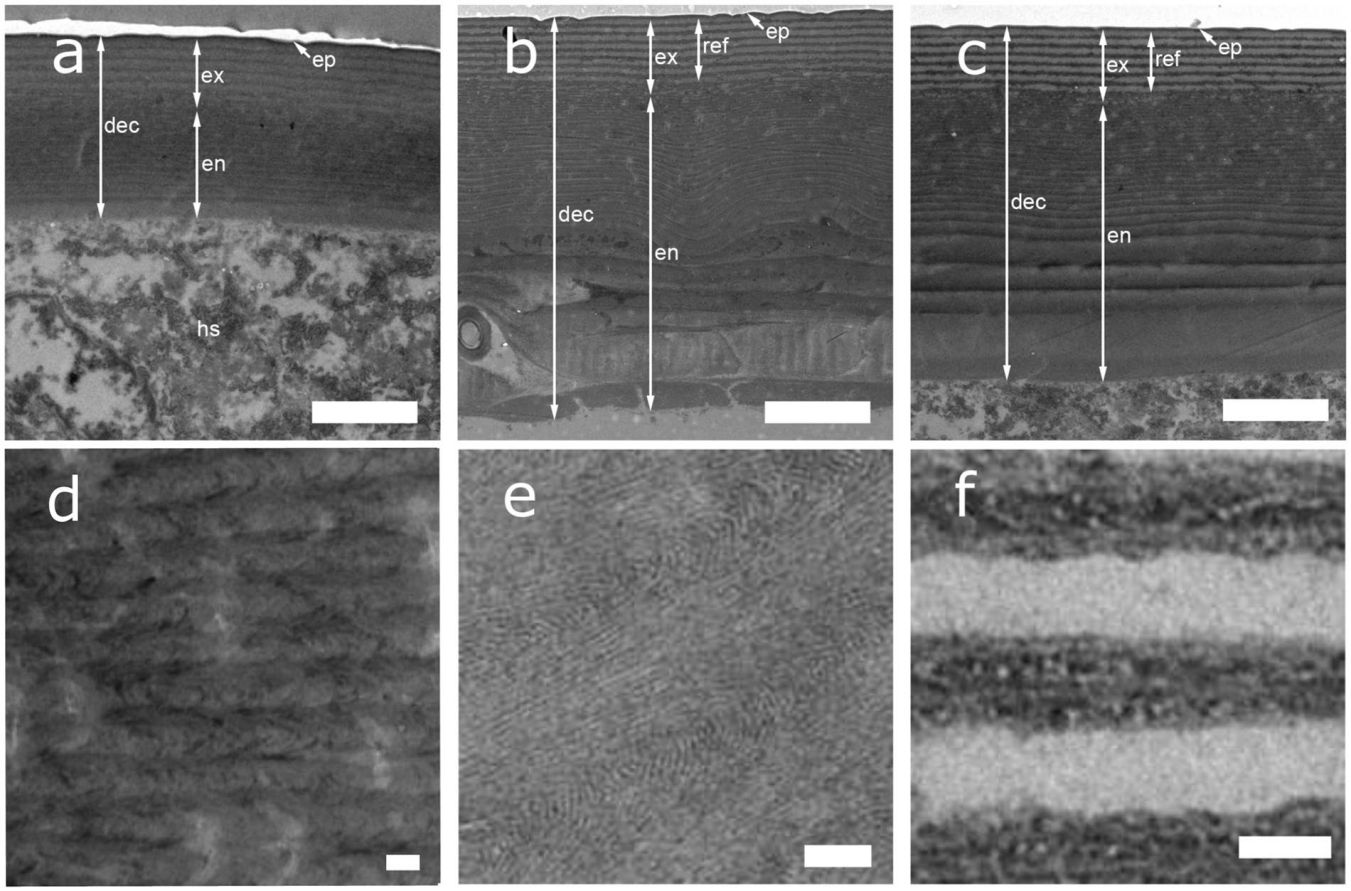
Figure 3 TEM sections of the developing dorsal elytral cuticle (“dec” in the figure), which illustrate the development of the endocuticle (“en”), exocuticle (“ex”), and epicuticle (“ep”). The cuticle after ecdysis (Stage 4: (**a**)) is considerably thinner than in the later stages 5 and 6 (**b**,**c**). The hemolymph space (“hs”) is visibile underneath the elytra. In (**b**), the young imago shows a more defined exocuticular multilayer reflector (“ref”) and a thicker endocuticle. The old imago, (**c**) exhibits a fully-formed cuticle. At higher magnification, it is possible to appreciate the structure of the inner exocuticle in (**d**,**e**) and the alternating fibril arrangement in the multilayer reflector in (**f**) for the fully formed imago. Scale bar: 2 *μ*m for (**a**–**c**), and 100 nm for (**d**–**f**).

In order to resolve the cuticular ultrastructure, we image the last stages of the development using TEM and examine an individual immediately after ecdysis (stage 4), a young imago (stage 5; 5 days after emergence), and a fully developed imago (stage 6; 10 days after emergence). In stage 4 the dorsal elytral cuticle has an overall thickness of 1.0–1.5 *μ*m (Fig. 3a). The presumptive epicuticle appears to be formed by two thin layers with the innermost being more highly electron absorbing, (darker in the TEM images) than the outer one. The exocuticle consists of two distinguishable parts: an inner section of tightly packed layers arranged in a helicoidal fashion and outer section showing 5–7 alternating layers of varying electron densities, which constitute the multilayer reflector. At this stage, the endocuticle has not been deposited yet. The outer exocuticle in stage 5 appears thicker and more uniform (Fig. 3b) consisting of 15–20 alternating layers. The contrast between the alternating layers of the reflector is more pronounced. In Fig. 3d,e it is possible to observe a helicoidal organization of the chitinous fibres in the dark-contrasted area of the inner exocuticle. Furthermore, the first endocuticlar layers have been deposited. In stage 6, (Fig. 3c) the arrangement is similar to the earlier stage and only an increase in the number of endocuticular layers is observed. As far as the architecture of the chitin fibrils in cuticle is concerned, we distinguish two types of arrangements: pseudo-orthogonal endocuticular layers (Fig. 3f) and a helicoidal exocuticle (Fig. 3d,e). The pseudo-orthogonal one has been previously reported to be common in beetle endocuticle^36–38^ but does not exhibit macrofibres, as present in several beetle species^22^. In the helicoid we can recognize alternating layers of different electron density.

**Figure 4:**
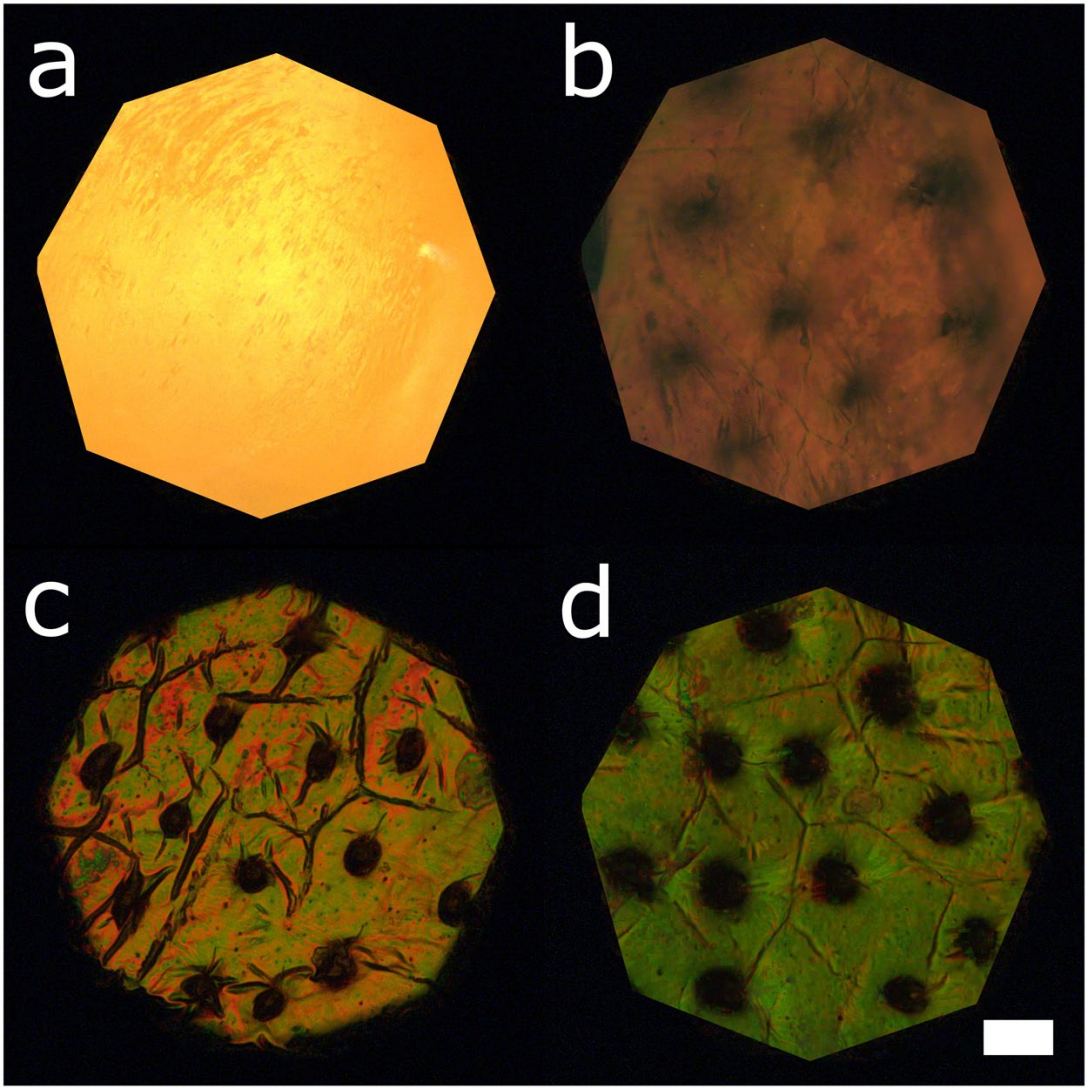
Micrographs of the developing cuticle. (**a**) shows the yellow pupal skin encasing the imago during pupation, when the cuticle is not yet formed. 3–4 days later (**b**), the adult cuticle is starting to form and is distinguishable underneath the pupal skin. The formation of trabeculae is evident from the presence of dark invaginations. In (**c**), the imago has emerged but the cuticle is still not fully expanded. 5 days after ecdysis (**d**), the cuticle has reached its final conformation. Scale bar: 1 mm.

**Figure 5:**
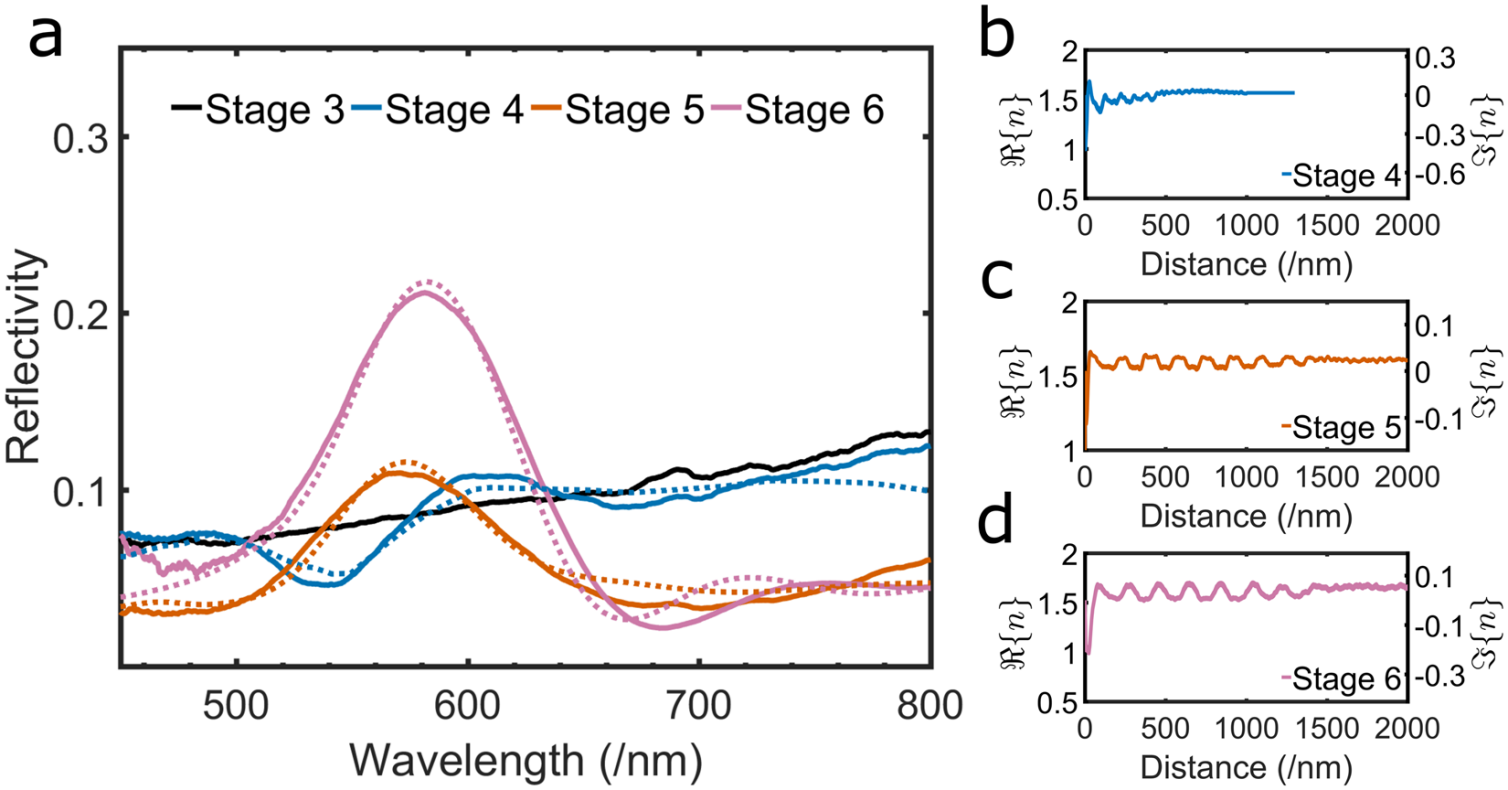
(**a**) The experimental spectra collected from a 10 *μ*m spot (solid lines) compared to the spectral response as predicted by the transfer-matrix simulations (dashed lines). (**b**–**d**) The real part and imaginary part of the refractive index as a function of the cuticle’s depth at different stages of development of the cuticle, see legend.

The development of the exocuticlar ultrastructure is monitored in living beetles spectroscopically. Figure 4a shows a micrograph in epi-illumination of the young pupal skin and the cuticle of the beetle in stage 4, 5, 6 is illustrated in Fig. 4b–d respectively. The spectral response for stages 3 to 6 is reported in Fig. 5a. By comparing the measured optical response with the one predicted from transfer-matrix calculations^35^ (Fig. 5a), we can quantify the variations of the cuticle in terms of its composition.

The independent variables used in transfer-matrix method are: (i) the dimensions of the layers constituting the exocuticle and (ii) their corresponding refractive indexes. To evaluate (i) for each stage of growth we extract the thickness of the layers from the TEM sections (see Supplementary Information). The refractive index of the chitin-protein matrix *n*_1_ used for this study is the one measured in refs 39, 40 for all the stages while the refractive index of the pigmented layers *n*_2_ is calculated using the effective medium approximation for each stage, since the ratio between the chitin-protein matrix and pigments changes during the development, by least squares minimisation of the difference between the predicted and experimental reflectivity. The value of real and imaginary part of the refractive indexes are shown in Fig. 5b–d. In the adult stage we assume that the only pigment present in the exocuticle is melanin, as reported in ref. 41, while during ecdysis a mixture of quinones and melanin is also considered (see Supplementary Information for the full discussion).

The young pupal skin (Fig. 4a) does not show any evidence of structural colour as its spectrum is typical for pigment absorption (Fig. 5a). Nevertheless, this incoherent pigmentary layer is still present immediately before the ecdysis (Fig. 4b) and it plays a filtering role in the measured spectrum^42^. By absorbing in the 400–500 nm region, the pupal skin decreases the signal produced by the developing multilayer. Hence, the reflectivity in this part of the spectrum is lower than the one predicted from the modelling of the Bragg stack only (Fig. S2).

After ecdysis, we observe a strong drop in reflectivity in the 530–550 nm wavelength region (Fig. 5a) which can be explained including in the calculation the presence of quinones in addition to melanin. These compounds have been shown to play a role in melanogenesis (which takes place during the sclerotisation of the cuticle, as reported in refs 43–45). The presence of quinones is further confirmed by the reddish colouration observed in the microscopic image of the cuticle in stage 4 (Fig. 4b), as expected in presence of this class of compounds^46, 47^. In particular, we conclude by fitting the measured spectra with our model that the relative amount of melanin and quinones in the pigmented layers in stage 4 is 15% and 5%, respectively. By comparing stage 4 with 5 and 6, we also note that the multilayer is yet not fully developed at this stage: by plotting the complex refractive index as a function of the multilayer’s depth, we observe that the contrast between the layers is low and that the distance between them is small (Fig. 5b–d).

In stage 5 the cuticle shows a broad reflectivity peak around 580 nm (Fig. 5a). At this stage, quinones are not present and the measured spectra agrees with the calculations using a melanin content of 57% for the pigmented layers. Moreover, the layering is very well defined and the distance between layers has substantially increased with respect the other stages (Fig. 5c).

Finally, when the cuticle of the old imago is fully expanded (Fig. 4d), the peak reflectivity is considerably stronger than the one of the younger imago (Fig. 5a). At this stage the melanisation has completed and we estimate that the final percentage of melanin contained in the pigmented layers of chitin-protein matrix is 79% (Fig. 5d).

## Conclusion

Our multi-scale imaging and spectroscopical study allowed us to reveal the different processes involved in the development of the exocuticule of a structurally coloured beetle.

We observed that the adult colouration of this species is achieved by an increase in the dimensions of the layers and by their melanisation in the outer exocuticle. Interestingly, the layer deposition and the melanisation happen in different stages. It is particularly surprising that the latter process is observed after stage 4 when the deposition of the endocuticle has already started. Therefore, even small variations during the development can result in extreme colour differences, making these architectures suitable for quick adaptation and species diversification - possibly faster than changing highly conserved biosynthetic pathways for pigmentation, which are controlled by a complex set of enzymes and genetics^48^, including different gene classes for biosynthesis and spatiotemporal positioning of pigments^49^. In comparison, altering cuticle deposition patterns appears to be a simpler approach. This is exemplified by the fact of numerous repeated evolutionary origins of multilayer reflectors in beetles^18^ and also explains how closely related species, such as in the beetle genera *Eupholus* and *Cetonia*, can produce very different colourations.

High resolution TEM images reveal that a helicoidal organization of chitin fibrils is maintained in the entire cuticle^37^. However, even if the characteristic dimensions of such helicoidal architecture do not contribute to the optical response in this case, its dimensions (of about 800 nm) might indicate that exocuticle is predisposed to the development of chiral Bragg reflectors^18^.

More generally, this study shed new insight on cuticle development: from an evolutionary point of view, it is interesting to note that pigmented-based multilayer reflectors are very common among different insects and other arthropods and therefore the mechanisms observed in this case of development might be generalized to other species, since the same materials/strategies are involved.

## Methods

Adult *G*. *viridula* beetles were collected from the meadows by the river Cam in Cambridge, England (52°12′41.2″N, 0°07′43.4″E) and reared in the lab as described in ref. 50.

### Synchrotron microtomography

Dead beetles at different stages were immersed in an EtOH 70% aqueous solution and scanned at the TOPO-TOMO beamline^51^ of the ANKA Synchrotron Radiation Facility at KIT, Germany. 3,000 radiographic projections covering an angular range of 180° were acquired using a filtered polychromatic beam with the spectral peak at about 15 keV. An indirect detector system composed of a 12 *μ*m LSO:Tb scintillator^52^, diffraction limited optical microscope (Optique Peter) and 12 bit pco.dimax high speed camera with 2016 × 2016 pixels resolution^53^ was employed to capture the frames with an exposure time of 16.6 ms each, resulting in an overall scan duration of 49.8s. A 5x optical magnification led to an effective pixel size of 2.44 *μ*m. Tomographic reconstruction was performed with the GPU-accelerated filtered back projection algorithm implemented in the software framework UFO^54^. Volume renderings of tomographic data were performed with Drishti 2.5.

### Transmission electron microscopy

TEM sections were prepared according to the following procedure: first of all, fixation was initiated by immersing the elytra in a buffer containing glutaraldehyde (2 wt%), formaldehyde (2 wt%) and sodium cacodylate (0.05 M) at pH 7.4 for 18 hours at 4 °C. Then the elytra were rinsed five times with deionized water (DIW) and fixed for 48 hours at 4 °C in a second buffer containing osmium ferricyanide (1 wt%) and sodium cacodylate (0.05 M) at pH 7.4. Next, they were rinsed again in DIW and dehydrated in an ascending series of ethanol solutions from 50 wt% to 100 wt% dry ethanol. The next step was to bulk stain them with magnesium uranyl acetate (3 wt%) in pure dry ethanol for 48 hours at 4 °C in the dark. Afterwards, the elytra were rinsed 5 times in pure dry ethanol and repeatedly frozen and thawed in liquid nitrogen. This procedure was repeated 10 times in order to facilitate the subsequent infiltration with epoxy resin. The elytra were rinsed 5 times in dry ethanol (100 wt%), twice in dry acetone and 3 times in dry acetonitrile. They were incubated overnight in a 50/50 mixture of acetonitrile and Quetol 651 epoxy resin (without the catalyst BDMA) in uncapped tubes to allow the acetonitrile to gradually evaporate. They were subsequently placed in fresh Quetol (without BDMA) every day for 2 weeks and subsequently in Quetol 651 (with BDMA) every day for a further 2 weeks. In order to perform vertical sectioning, the elytra were orientated in a coffin mould and the resin was left to cure for a minimum of 48 hours at 65 °C. Finally, the elytra were sectioned at 50–60 nm with a Leica UCT ultramicrotome using a 35° wedge angle diamond knife (Diatome Ltd) and mounted on 300 mesh copper grids with a carbon film (EM Resolutions Ltd) for imaging. They were viewed in a FEI Tecnai G2 operated at 200 kV (camera: AMT XR60B; software: Deben).

### Optical microspectroscopy

Optical microscopy was performed using a Zeiss Axio.Scope optical microscope in Köhler illumination equipped with a 20X objective (Zeiss EC Epiplan-APOCHROMAT 0.6 HD DIC) coupled to a spectrometer (Avantes HS2048) via an optical fibre (Thorlabs, FC-UV100-2-SR). This enabled us to collect the spectra from a circular spot (diameter: 10 *μ*m). 20 spectra were collected for each sample using an integration time of 500 ms and normalized with respect to a protected silver mirror (Thorlabs, PF10-03-P01). These experimental spectra were then compared to those originated from a transfer-matrix simulation. The simulation was based on the open source Python code developed in ref. 55. Both the real and imaginary part of the refractive index were assumed to be proportional to the optical density derived from the TEM images by converting the multilayer image to grey levels averaged using ImageJ^56^. The TEM images were rescaled as described in the Supplementary Information in order to account for the small deformations that occurred during the sample preparation. The refractive indices accounted for dispersion. 20 such profiles with a width of 500 nm were used as input for the model which was run for light incident from −37° to 37°. The spectra obtained were then averaged together. In this way, we replicated the experimental conditions in which the spectra were then collected from a 10 *μ*m spot with a numerical aperture of 0.6.

### Data availability

All the research data supporting the publication are available from the University of Cambridge data repository (https://doi.org/10.17863/CAM.8829).

## Electronic supplementary material

Supplementary Information

SI Video 1

SI Video 2
