# Supplementary material for: Development of structural colour in leaf beetles
Source: Sci Rep. 2017 May 2;7:1373. doi: 10.1038/s41598-017-01496-8 (PMC5430951; doi:10.1038/s41598-017-01496-8)
Supplement: Supplementary file 1 — Supplementary Information [file 41598_2017_1496_MOESM1_ESM.pdf]

Supplementary information: Development of structural colour in leaf beetles

Olimpia D. Onelli[1], Thomas van de Kamp[2], Jeremy N. Skepper[3], Janet Powell[3], Tomy dos Santos Rolo[4], Tilo Baumbach[2,4], and Silvia Vignolini[1,*]

[1]Department of Chemistry, University of Cambridge, Lensfield Road, Cambridge, CB2 1EW, UK
[2]Laboratory for Applications of Synchrotron Radiation (LAS), Karlsruhe Institute of Technology (KIT), Kaiserstr. 12, D-76131 Karlsruhe, Germany
[3]CAIC, Anatomy Building, Cambridge University, Downing Street, Cambridge CB2 3DY, UK
[4]Institute for Photon Science and Synchrotron Radiation (IPS), Karlsruhe Institute of Technology (KIT), Hermann-von-Helmholtz-Platz 1, D-76344 Eggenstein-Leopoldshafen, Germany
[*]sv319@cam.ac.uk

ABSTRACT

Structural colours in living organisms have been observed and analysed in a large number of species, however the study of how the micro- and nano-scopic natural structures responsible of such colourations develop has been largely ignored. Understanding the interplay between chemical composition, structural morphology on multiple length scales, and mechanical constraints requires a range of investigation tools able to capture the different aspects of natural hierarchical architectures. Here, we report a developmental study of the most widespread strategy for structural colouration in nature: the cuticular multilayer. In particular, we focus on the exoskeletal growth of the dock leaf beetle *Gastrophysa viridula*, capturing all aspects of its formation: the macroscopic growth is tracked via synchrotron microtomography, while the submicron features are revealed by electron microscopy, light spectroscopy combined with numerical modelling. In particular, we observe that the two main factors driving the formation of the colour-producing multilayers are the polymerization of melanin during the ecdysis and the change in the layer spacing during the schlerotisation of the cuticle. Our understanding of the exoskeleton formation provides a unique insight into the different processes involved during metamorphosis.

Numerical simulation

For each pixel in the TEM images, the grey level and position were measured using Image J (each line profile was averaged over 500 nm of cuticle) and then loaded into the Python simulation. During the TEM sample preparation, the cuticle expanded (or contracted, for the adult stage) by a small amount and therefore the multilayer dimensions have been corrected to match the central reflectance peak, as summarized in table 1.

We believe that the younger stages have a greater water content and are expected to shrink after removal from the insect. In the adult insect (stage 6), the cuticle is already dead and hardened therefore it does not shrink after removal. However, during processing for TEM we expect a little shrinkage or swelling in the primary fix if the effective molarity is not perfectly isotonic. The correction has been estimated independently by matching the peak wavelength for the simulated spectra to the experimental data.

Description	Correction
4. Imago immediately after ecdysis	- 15.0 %
5. Imago before cuticular expansion	- 9.4 %
6. Imago with fully developed cuticle	+ 5.0 %

Table 1. Summary of the size correction applied to the TEM images.

The dispersion relations for the chitin-protein matrix refractive index n_1 used in this study was:

$$n_1 = 1.5145 + 8800/\lambda^2, \tag{1}$$

where λ is the wavelength of the incident light (in nm). For the melanin-contain layers the refractive index n_m was assumed to be:

$$n_m = 1.648 + 23700/\lambda^2 + 0.56 * \exp(-\lambda/270)i. \tag{2}$$

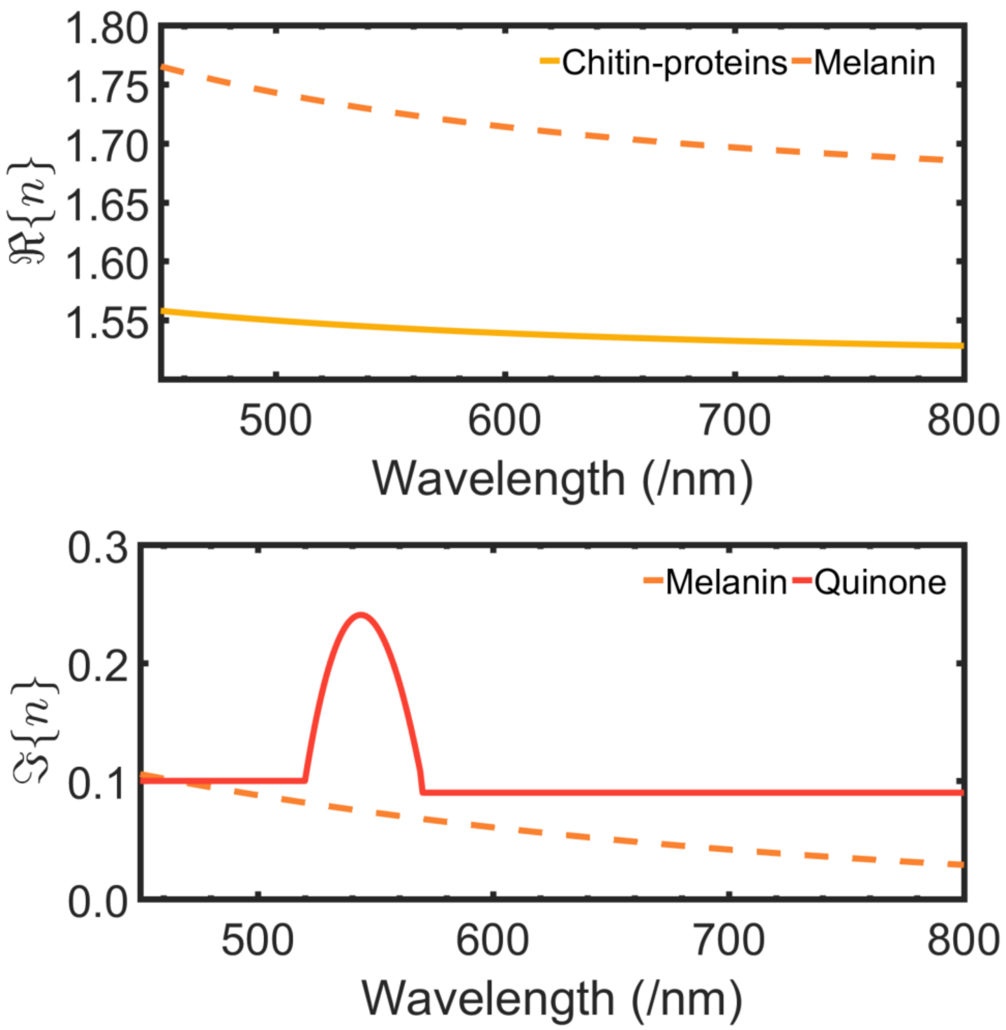

Figure 1. The dispersion relations for the chitin-protein matrix and melanin-containing layers (top, equation 1 and 2) and for the imaginary part of the melanin-containing layers and quinone-containing ones (bottom, equation 2 and 5).

For the quinone-containing layers, n_q:

$$\begin{aligned} n_q &= 0.1i \text{ for } \lambda < 510 \text{ nm} && (3)\\ &= 0.009i \text{ for } \lambda > 589 \text{ nm} && (4)\\ &= -19936643/\lambda^2 + 73331\lambda - 67 \text{ elsewhere.} && (5) \end{aligned}$$

The total refractive index n_2 for the pigment-containing layers was calculated using the effective medium approximation:

$$n_2 = n_1 f_1 + n_m f_m + n_q f_q, \quad (6)$$

where f_1, f_m,f_q are the relative filling fractions for the chitin-protein matrix, melanin, and quinone respectively. Hence,

$$f_1 + f_m + f_q = 1. \quad (7)$$

The dispersion relations are illustrated in Fig. 1.

The initial medium was assumed to be air (n = 1.00029, infinite thickness) while the final medium was assumed to be the same as the last point measured (infinite thickness). The s and p polarisations were calculated separately

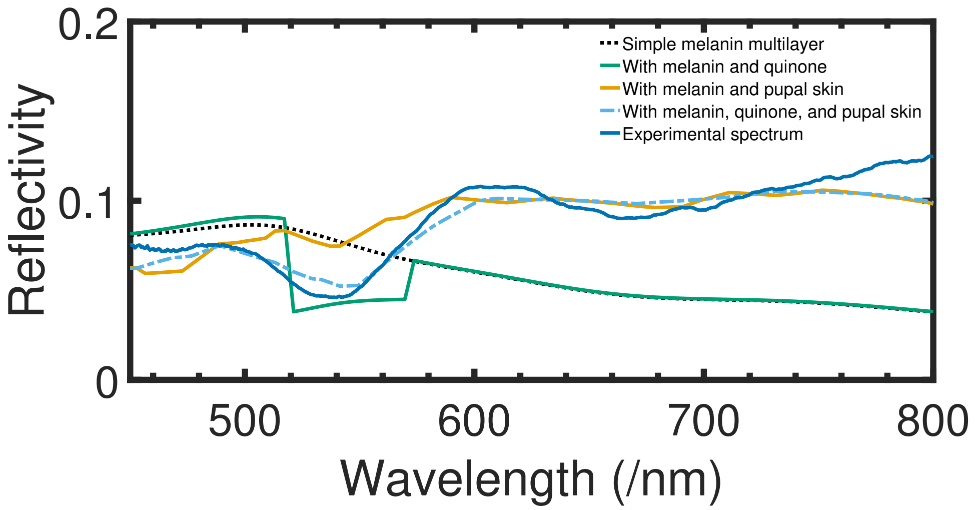

Figure 2. The experimentally measured as compared to the predicted intensity from the numerical model. See legend.

and then averaged together to represent unpolarised light. The simulation run between $\lambda = 400$ nm and $\lambda = 800$ nm in steps of 1 nm. The incident angle was varied between -37° and +37° in 2-degree steps to match the numerical aperture of the microscope objective. The result from each calculation was averaged together: the Köhler illumination set-up guaranteed a uniform illumination and hence we were able to perform a linear average.

Stage 4 simulation

For comparison, in Fig. 2 we show how the predicted spectra varies if:

- we assume that no quinones are present;
- we assume that the yellow pupal skin does not filter the signal;

our analysis shows that the effect of quinones must be included in order to explain the drop in the reflectance between 500 and 600 nm.

Supplementary video legends

Supplementary video 1: *G. viridula* larvae molting to pupae. The footage covers a time span of 6 hours. **Supplementary video 2:** *G. viridula* metamorphosis: from pupae to imagoes. The footage covers a time span of 12 hours.
